# Mechanisms of Nonvesicular Ceramide Transport

**DOI:** 10.1177/25152564231208250

**Published:** 2023-10-17

**Authors:** Lena Clausmeyer, Florian Fröhlich

**Affiliations:** 1 Department of Biology/Chemistry, Bioanalytical Chemistry Section, 9186 Osnabrück University, Osnabrück, Germany; 2 Center of Cellular Nanoanalytics Osnabrück (CellNanOs), 9186 Osnabrück University, Osnabrück, Germany

**Keywords:** ceramide, transport, ceramide transport protein, Nvj2, Svf1, tricalbins

## Abstract

Ceramides, as key components of cellular membranes, play essential roles in various cellular processes, including apoptosis, cell proliferation, and cell signaling. Ceramides are the precursors of all complex sphingolipids in eukaryotic cells. They are synthesized in the endoplasmic reticulum and are further processed at the Golgi apparatus. Therefore, ceramides have to be transported between these two organelles. In mammalian cells, the ceramide transfer protein forms a contact site between the ER and the trans-Golgi region and transports ceramide utilizing its steroidogenic acute regulatory protein-related lipid transfer domain. In yeast, multiple mechanisms of nonvesicular ceramide transport have been described. This involves the nuclear–vacuolar junction protein Nvj2, the yeast tricalbin proteins, and the lipocalin-like protein Svf1. This review aims to provide a comprehensive overview of nonvesicular ceramide transport mechanisms and their relevance in cellular physiology. We will highlight the physiological and pathological consequences of perturbations in nonvesicular ceramide transport and discuss future challenges in identifying and analyzing ceramide transfer proteins.

## Introduction

Ceramide, an essential lipid molecule, serves as the fundamental building block for complex sphingolipids and plays a pivotal role in cellular signaling pathways and the regulation of cell death. Dysregulated levels of ceramide have been implicated in the pathogenesis of various neurodegenerative disorders, diabetes, and cardiovascular diseases (Boon et al., 2013; Mielke et al., 2013; Hilvo et al., 2020). Therefore, gaining a comprehensive understanding of ceramide metabolism and its transport between organelles is very important. Here, we will review the current knowledge surrounding ceramide transfer proteins.

**Figure 1.**
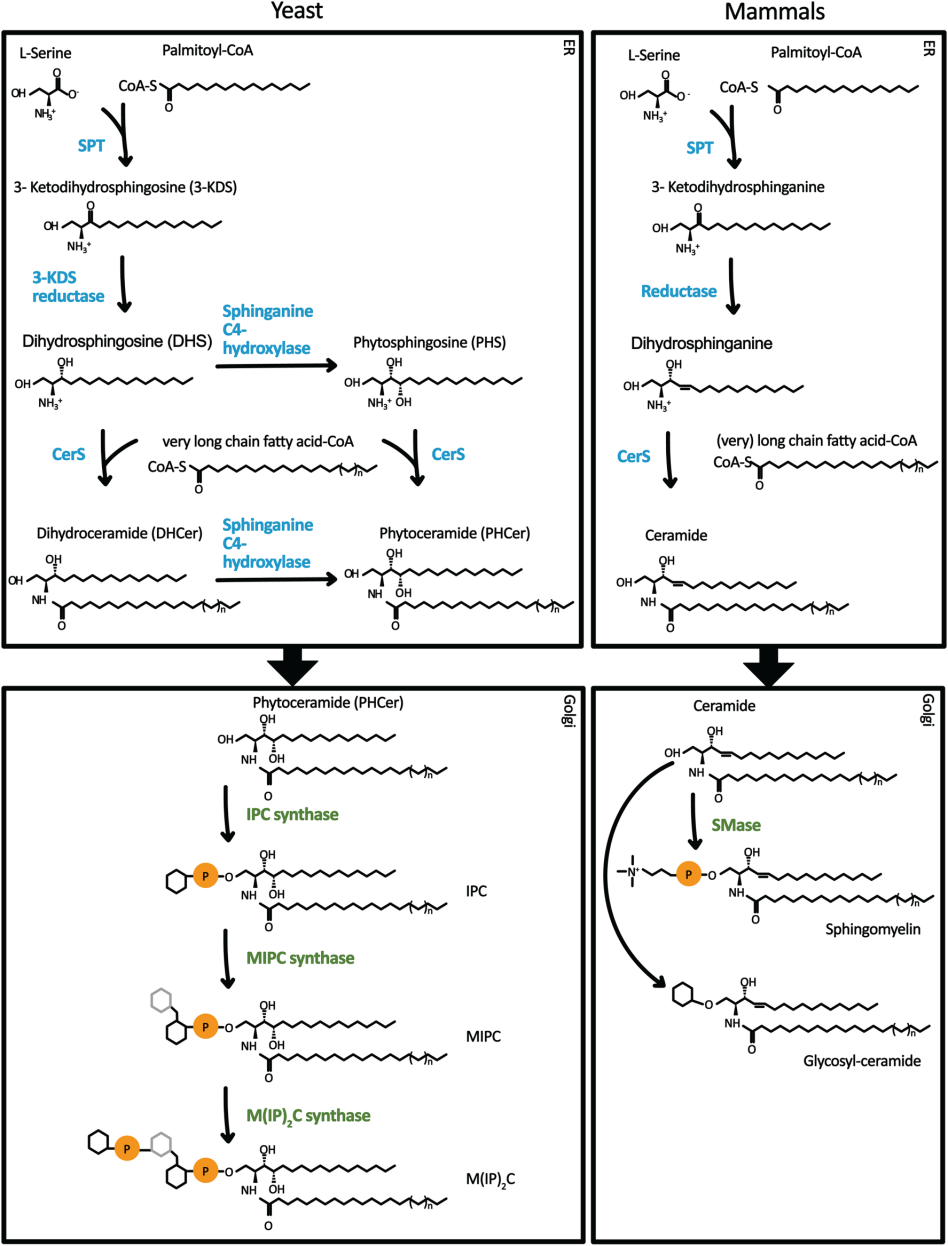
Overview of sphingolipid metabolism. A schematic representation of the sphingolipid metabolism in yeast and mammals is depicted. The metabolic pathways are outlined, highlighting the sequential steps through which sphingolipids are synthesized and metabolized within the cell. Key enzymes and their corresponding genes responsible for catalyzing the different reactions are depicted, along with the intermediate molecules formed at each stage. The sspatial distribution of these enzymatic reactions within the cellular compartments is illustrated, emphasizing the significance of inter organelle transport of ceramide. For simplicity, only sphingomyelin and glucosyl-ceramide are shown as complex sphingolipids in mammalian cells.

Ceramide is synthesized at the endoplasmic reticulum (ER). The first and rate-limiting step in sphingolipid biosynthesis is the condensation of serine and palmitoyl coenzyme A at the cytosolic leaflet of the ER membrane by the serine palmitoyl transferase (SPT) (Hanada, 2003; Lowther et al*.*, 2012). The resulting product, three keto-sphinganine is rapidly reduced to dihydrosphingosine (DHS) by the three keto-dihydrosphingosine reductase (Figure 1). The ceramide synthase (CerS) amide links DHS with a fatty acid to produce ceramide, the backbone of all complex SPs (Hannun and Obeid, 2018). This reaction occurs in the ER membrane. In yeast, the CerS consists of two catalytic subunits Lac1 and Lag1, and one accessory subunit Lip1 (Guillas et al., 2001; Schorling et al., 2001; Vallee and Riezman, 2005). While Lac1 and Lag1 are nonessential and able to compensate for the loss of each other, Lip1 is essential for CerS activity, but its function is unknown (Jiang et al., 1998; Barz and Walter, 1999; Schorling et al., 2001). In mammals, ceramide is synthesized by six different ceramide synthases (CerS1-6). Each enzyme prefers a defined length of fatty acyl-CoA, for example, CerS2 incorporates mainly C22-24-CoAs into ceramides while CerS4 prefers C18-C20-CoA (Laviad et al., 2008; Tidhar et al., 2012, 2018). In yeast, mainly VLCFA-CoA with a length of 26 carbon atoms is used. In the Golgi apparatus, headgroups are attached to ceramide, yielding complex SPs and glycosphingolipids. Consequently, ceramide needs to be transported from the ER to the Golgi. In both, mammalian and yeast cells the rate-limiting SPT is regulated by ER ceramide levels further highlighting the importance of its transport out of the ER (Davis et al., 2019; Schäfer et al., 2023; Xie et al., 2023). In yeast, it has been described that up to 80% of the ceramides are transported to the cis*-*Golgi via coat protein complex II (COPII)-coated vesicles (Funato and Riezman, 2001; Sato and Nakano, 2007; Miller and Barlowe, 2010). In mammalian cells, only VLCFA-containing ceramides are hypothesized to be transported by vesicular transport to the Golgi apparatus. However, in this review, we will focus on the known mechanisms of nonvesicular ceramide transport.

## Nonvesicular Transport of Lipids

Proteins synthesized in the ER are primarily transported via vesicles to the cis-Golgi for sorting and further directed to specific organelles. Consequently, lipids necessary to form membrane vesicles also utilize the same transport machinery. However, to maintain lipid homeostasis and organelle-specific composition, lipids are also transported individually by lipid transfer proteins (LTPs) (Wong et al., 2019). These proteins operate at membrane contact sites (MCSs) where two membranes are in close proximity, up to 30 nm apart (Helle et al., 2013). LTPs reside at various MCSs, such as between the ER and plasma membrane (PM), ER and endosome, ER and vacuole/lysosome, and ER and Golgi (Eisenberg-Bord et al., 2016). This nonvesicular transport occurs either within the concentration gradient of specific lipids or against it, requiring energy. One well-described LTP is the oxysterol-binding protein (OSBP) (Mesmin et al., 2013). OSBP transports sterols from the ER to the trans-Golgi where it is exchanged for phosphatidylinositol-4-phosphate which is subsequently transported to the ER. In the ER, PI4P is hydrolyzed to PI by the PI4P-phosphatase Sac1 (Beh et al., 2001; Levine and Munro, 2001; Raychaudhuri and Prinz, 2010; Stefan et al., 2011; Zewe et al., 2018).

## Ceramide Transfer Proteins in Mammalian Cells

The ceramide transport protein (CERT) plays a critical role in the intracellular transportation of ceramides from the ER to the Golgi in mammals. This cytosolic protein, with a size of 68 kDa, functions by collecting newly synthesized ceramides at the ER and facilitating their transfer to the trans-Golgi (Hanada et al., 2003; Kawano et al., 2006; Mizuike et al., 2023). CERT encompasses various functional domains and motifs, similar to other lipid transfer proteins (LTPs). The C-terminal domain, known as the steroidogenic acute regulatory protein-related lipid transfer (START) domain, is essential for ceramide binding. Within the CERT START domain lies a long amphiphilic cavity ideally suited to pick up one ceramide from donor membranes and release it to acceptor membranes, with specific amino acids at the cavity's tip interacting with the ceramide's amide and hydroxyl groups (Kudo et al*.*, 2008). This hydrophobic cavity is believed to function as a molecular measuring device, akin to the bacterial membrane acyltransferase, PagP (Ahn et al*.*, 2004). Interestingly, CERT exhibits a higher affinity for ceramides containing 14–20 carbon atoms compared to those with longer acyl chains (C22–C24) (Kumagai et al*.*, 2005).

The N-terminus of CERT comprises a Pleckstrin Homology (PH) domain, which specifically binds to phosphatidylinositol 4-monophosphate (PI(4)P) primarily found in the trans-Golgi, thus anchoring the protein to the trans-Golgi (De Matteis et al., 2005). Another important motif is the “two phenylalanines in an acidic tract” (FFAT) motif, which interacts with ER resident VAP family proteins, establishing a bridge-like connection between the ER and Golgi (Kawano et al., 2006). CERT's activity is governed by the serine-repeat motif (SRM), located close to the PH domain. Dephosphorylation of this motif renders CERT active, enabling the transport of ceramides from the ER to the trans-Golgi, while phosphorylation results in inactivity and no ceramide transport. Casein kinase 1 plays a key role in regulating CERT activity, responsible for its inactivation (Kumagai and Hanada, 2019).

Mutations in CERT are linked to the development of intellectual disability syndrome. The SRM motif is a frequent site for mutations that render the protein unresponsive to inactivation by phosphorylation (Murakami et al., 2020; Tamura et al., 2021). Another recent study has identified an additional domain as a hot spot for the occurrence of mutations. This domain was described as the central core domain (CCD). The CCD harbors two coiled-coil structures and may function as a dimer interface for dimeric CERT arrangement (Gehin et al., 2023). Other studies have described a trimeric arrangement of CERT (Revert et al*.*, 2018; Goto et al*.*, 2022). How the protein switches between monomeric, dimeric, and trimeric states, and which ones affect CERT function, remains to be revealed in the future. However, all identified mutations so far result in higher CERT activity, causing an increased ceramide flux to the trans-Golgi and correlating with lower ceramide concentration in the ER membrane. This most likely reduces SPT inhibition, leading to a concomitant increase in de novo sphingolipid synthesis. It also impairs glycosphingolipid production at the Golgi and the production of dihydrosphingolipids, which can contribute to neuropathology.

## Ceramide Transfer Proteins in Yeast Cells

The majority of ceramides in mammalian cells are transported by CERT. As previously mentioned, ceramides in yeast cells are transported through both vesicular and non-vesicular mechanisms. Concerning the nonvesicular ceramide transport pathways in yeast, Nvj2-dependent ceramide transport, tricalbin-dependent ceramide transport, and Svf1-dependent ceramide transport have been described.

Nvj2 is a component of the nuclear vacuolar junction, an MCS between the nuclear ER and the vacuole in yeast cells. It possesses one transmembrane domain embedded in the ER membrane, with the remaining part of the protein protruding into the cytosol. Notably, Nvj2 shares a common structural feature with the CERT protein—the PH domain, which also binds to PI4P. Additionally, Nvj2 contains a synaptotagmin-like mitochondrial lipid-binding protein (SMP) domain that potentially facilitates ceramide transport (Toulmay and Prinz, 2012; Liu et al*.*, 2017). Furthermore, other studies describe SMP domains as lipid transfer domains (Schauder et al*.*, 2014; AhYoung et al*.*, 2015; Yu et al*.*, 2016).

Recent findings have demonstrated that under ER stress conditions, Nvj2 localizes to the ER-Golgi MCSs and the medial-Golgi, thereby promoting tethering of the MCS and supporting ceramide transport from the ER to the medial-Golgi (Liu et al*.*, 2017). The authors show both, a reduction in complex sphingolipids with a concomitant increase in ceramides. In addition, mutations in the SMP abolish these phenotypes, supporting the direct role of Nvj2 in ceramide transport. However, under nonstress conditions, Njv2 does not localize within the ER-Golgi MCS, yet ceramide transport to the Golgi still occurs when the secretory pathway is blocked in *nvj2Δ* cells. These observations suggest that other pathways may be involved in ceramide transport (Kopec et al., 2010; Toulmay and Prinz, 2012; Liu et al., 2017).

One potential pathway is mediated by the tricalbin proteins that have been described as potential ceramide transfer proteins between the ER and the medial Golgi (Ikeda et al*.*, 2020). The tricalbins (Tcb1, Tcb2, and Tcb3) share homology with the extended synaptotagmins (E-Syts) that play a role in the formation of MCSs (Giordano et al., 2013; Yu et al., 2016; Bian et al., 2018). Tricalbins possess a hydrophobic N-terminus forming a hairpin motif that anchors to the ER membrane (Hoffmann et al., 2019). This domain senses membrane curvature and prefers membranes with high curvature. To establish a bridge-like connection between two organelles the protein contains also multiple C2 domains that bind membranes in the presence of cytosolic Ca^2+^ (Schulz and Creutz, 2004; Hoffmann et al., 2019). Additionally, they also feature an SMP domain, suggesting a potential role in lipid transfer (Alva and Lupas, 2016; Wong and Levine, 2017). Various studies have associated these proteins with tethering functions at MCSs, either between the cER and PM or between the ER and medial-Golgi (Manford et al., 2012; Toulmay and Prinz, 2012; Collado et al., 2019; Hoffmann et al., 2019; Ikeda et al., 2020; Qian et al., 2021). The latter is proposed to occur during ER stress and promotes nonvesicular ceramide transport from the ER to the Golgi for complex SP synthesis. In *tcb1Δ*, *tcb2Δ*, and *tcb3Δ* cells, the ER–Golgi contact is reduced and acyl ceramide levels are increased. Acylceramide can be stored in lipid droplets (LDs) (Voynova et al., 2012). Its accumulation seems to stimulate LD formation (Ikeda et al., 2020).

Whether Nvj2 or the tricalbins directly transport ceramide with their SMP domains or promoting the MCS establishment, thus supporting indirect nonvesicular ceramide transport from the ER to the Golgi is still unclear and further investigation is required to elucidate their specific role. Whether the mammalian Nvj2 homolog Tex2 transports ceramides is currently unknown.

Finally, survival factor one (Svf1) was recently described as a potential ceramide transfer protein acting at the interface of the ER and the cis-Golgi (Limar et al., 2023). Svf1 is classified as a lipocalin protein. This protein family is known for its transport of small hydrophobic molecules such as steroids, fatty acids, retinoids, prostaglandins, and hormones (Flower, 1994; Chu et al., 1998). They are small circulatory proteins and their typically shape is given by an eight to ten-stranded antiparallel ß-sheet forming a ß-barrel closing itself via hydrogen bonds. Thereby it is flattened or elliptical in cross-sections. The internal ligand binding site is embedded in the tunnel-like structure and covered by a lid which is typically localized between the first two N-terminal ß-sheets. The lid can be either completely closed or partially opened (Flower et al., 1993; Flower, 1996). The family shows a low sequence homology, often below 20%, which makes the identification of clear homologs almost impossible. However, three large structurally conserved regions (SCRs) are found in nearly all proteins of the lipocalin family. The SCR1 includes the first strand and a 3_10_-like helix, the SCR2 localized within strands six and seven linked by a conserved loop and the SCR3 refers to strand eight and adjoining residues (Flower et al., 1991, 1993). One member of the lipocalin family, the tear lipocalin has been shown to directly interact with ceramide (Glasgow and Abduragimov, 2018).

**Figure 2.**
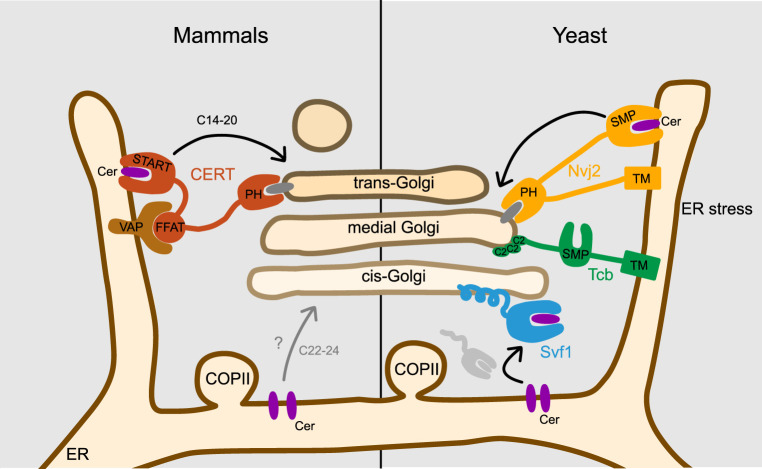
Model for the various ceramide transport pathways between the ER and the Golgi apparatus. The model depicts the CERT-mediated ceramide transfer in mammalian cells (left side) as well as some potential ceramide transport in COP-II vesicles. In yeast cells (right side), the potential mechanisms for Svf1-mediated ceramide transfer, tricalbin-mediated ceramide transfer, and the stress-induced Nvj2-dependent ceramide transfer between the ER and the Golgi are shown. Ceramides in yeast cells are also transported via COP-II-mediated transport. ER = endoplasmic reticulum; CERT = ceramide transport protein; COP-II = cis*-*Golgi via coat protein complex II.

Interestingly, Svf1 possesses two lipocalin domains, creating a hydrophobic cleft that enables it to accommodate ceramides based on molecular docking studies and targeted lipidomics. When *SVF1* is deleted, ceramides accumulate, and complex sphingolipids (SPs) decrease, suggesting that Svf1 acts as a transporter, shuttling ceramides between the endoplasmic reticulum and the Golgi apparatus. Svf1 localizes dynamically to the cis-Golgi apparatus and a cytosolic pool, and immune-purification of Svf1 reveals potential interaction partners from both the ER and Golgi organelles. For Golgi targeting, Svf1 relies on its N-terminal-AH helix and N-terminal acetylation, similar to other proteins targeting the Golgi via an AH (Behnia et al., 2007; Limar et al., 2023). Whether Svf1 acts as a monomer or in a multimeric form remains unknown. The proposed model suggests that Svf1 picks up ceramides from the ER, transiently interacts with it, and then targets the Golgi via its AH, releasing the ceramides for complex SP biosynthesis (Figure 2). The mechanism of ceramide release from the hydrophobic pocket of Svf1 remains uncertain, but one possible model involves exchange with another lipid at the Golgi apparatus, such as diacylglycerol (DAG), a product of complex SP biosynthesis. This is similar to CERT which has been shown to transport DAGs with lower affinity than ceramides (Kumagai et al., 2005). However, since ceramides are rapidly converted into complex SPs the abundance of DAG at the Golgi membrane could be sufficient.

### Future Directions and Concluding Remarks

**Figure 3.**
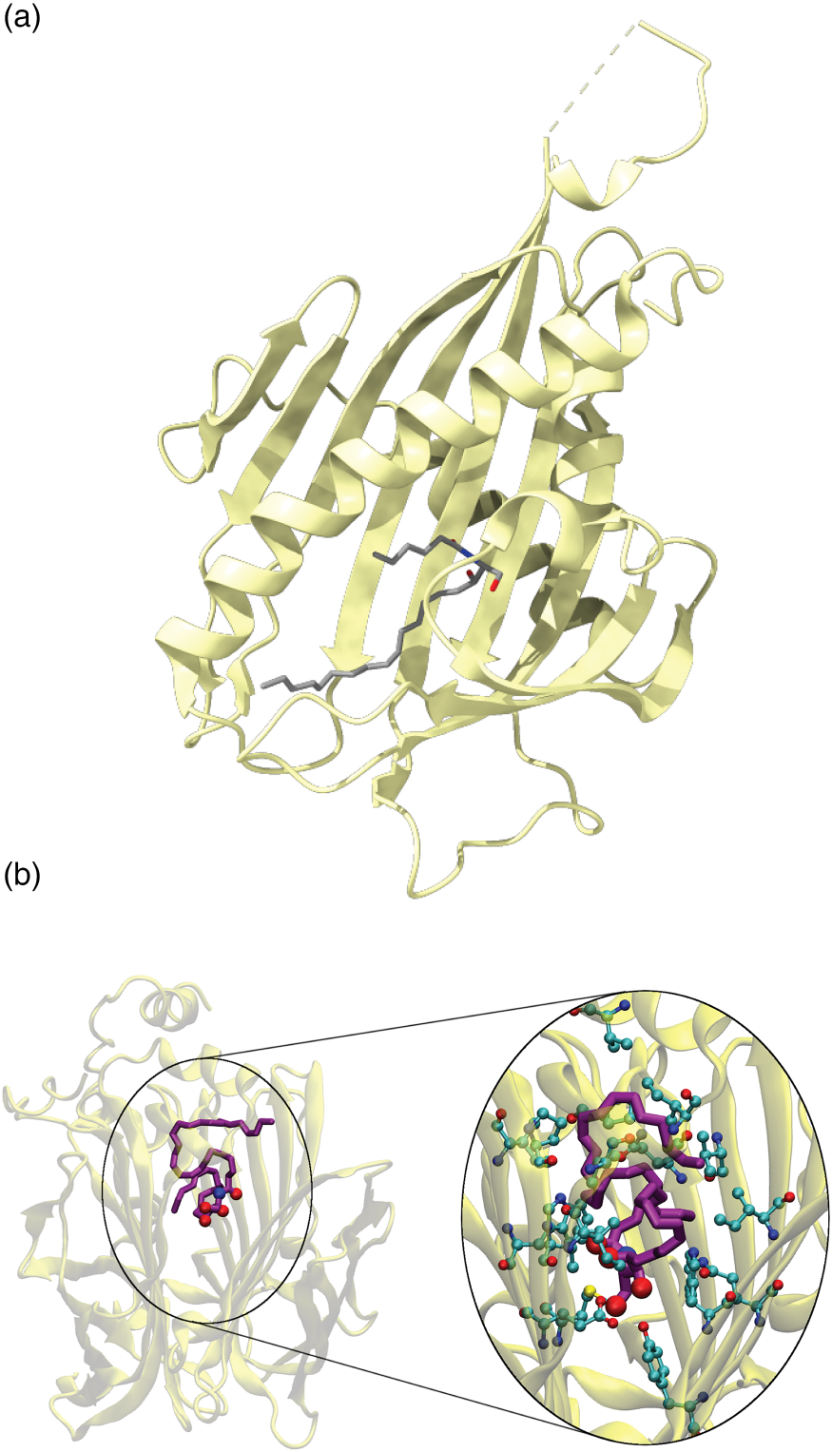
Analysis of the interaction of proteins and ceramides. (a) Crystal structure of the START domain of ceramide bound to C6: ceramide as a ligand (modified from PDB: 2E3N) (Kudo et al., 2008) shows the interaction of a hydrophobic pocket with the C6 cceramide (gray). (b) Molecular docking studies with C26 ceramide and the yeast protein Svf1 (Limar et al., 2023). A typical yeast 44:0;4 ceramide (purple) is modeled into the hydrophobic pocket between the two lipocalin domains of yeast Svf1. The AlphaFold prediction of Svf1 (AF-Q05515-F1) was used for the docking studies. ©2023 Limar et al. Originally published in Journal of Cell Biology https://doi.org/10.1083/jcb.202109162

For CERT, in vitro assays directly showing ceramide transfer have been established using liposome-based transfer assays (Hanada et al., 2003). Additionally, structural analysis revealed the direct binding of CERT to ceramide (Figure 3a) (Kudo et al., 2008). Furthermore, a photoactivatable and clickable ceramide (paCer) has been used to identify STARD7, along with CERT, as a ceramide-binding homolog, even though it lacks the transport activity of CERT (Bockelmann et al., 2018). Bi-functional and tri-functional ceramides and lipids are becoming more available and will allow the identification and study of ceramide transfer proteins in the future (Kol et al., 2019; Morstein et al., 2021). Importantly, ceramides harboring very long-chain fatty acids make in vitro studies, such as ceramide transfer assays and structural biology approaches, more challenging. VLCFA-containing ceramides are highly hydrophobic and difficult to solubilize. Thus, generating C26 ceramide-containing liposomes for direct ceramide transfer assays remains challenging. Consequently, it still remains unclear if VLCFA-containing ceramides in mammals are transported by a vesicular or nonvesicular pathway. In yeast, all ceramides and complex sphingolipids harbor VLCFAs. This might be one reason why most studies have used the levels of ceramides and complex sphingolipids as a readout for ceramide transfer (Liu et al., 2017; Ikeda et al., 2020; Limar et al., 2023). However, the levels of complex sphingolipids in yeast are also challenging to analyze by mass spectrometry-based lipidomics since no adequate standards for their quantification are commercially available. A major step forward in the analysis of ceramide transfer and binding proteins is also the structure prediction tools using AlphaFold (Jumper et al., 2021; Varadi et al., 2022). In combination with molecular dynamics simulations, this allows predictions of the binding mode of ceramides to proteins (Figure 3b). However, these predictions still require experimental validation using biochemical methods.
